# The Nurture of Royal Infants

**Published:** 1892-12-24

**Authors:** 


### THE NURTURE OF ROYAL INFANTS.

It is remarkable how often in English history the succession has been diverted from the reigning line by the death of all direct heirs. It almost seems as if, with some few exceptions, where our sovereigns have succeeded in rearing large families, a fate had pursued the Royal offspring. Such a fate, when the records are closely examined, did indeed pursue many of these unhappy little princes and princesses, but it will be found very frequently to consist of nothing more mysterious than dirt, bad management, and over physicking. To the dangers of the first item rich and poor were alike exposed, but the palace, less open to the winds of heaven, and provided, moreover, with additional disease traps under the name of luxuries, doubtless afforded a far deadlier atmosphere than the cottage. The plain mud floor of a country hovel, for instance, cannot have exhaled a noxious odour to be compared with that proceeding from the accumulation of rushes in the noble's hall, remaining sometimes undisturbed for many years, while a fresh sprinkling at the top gave a spurious air of cleanliness to the whole. So intolerable were the inconveniences arising from the filthy state of the apartments that the nobles were compelled to remove, at stated periods, from one country seat to another; these progresses (so Miss Strickland informs us) being elegantly termed "going to sweeten."

Katherine of Aragon, in her directions to the English governess of the Princess Mary, lays much stress on the observance of simple sanitary rules. "To have good respect to the cleanliness and well-wearing of her garments and apparel, both of her chamber and person, so that everything about her be pure, sweet, clean, and wholesome, as to so great a princess doth appertain; all corruptions, evil airs, and things noisome and unpleasant to be eschewed." The frequent plagues which the neglect of such observances entailed were by no means confined to the people. Low fever, probably some kind of typhoid, seized numberless victims from the Royal household; more virulent fevers, such as typhus, made periodic visits to the palace, and small-pox, on its introduction, became the most dreaded scourge of all, and was specially fatal to the children of the Stuart family. The practice of bleeding in the foot was then almost invariably resorted to for small-pox, and almost as invariably sealed the patient's fate.

But apart from epidemics to which all were subject, there were many dangerous stumbling-blocks in the way of the Royal infant. We read of cordials containing crushed pearls, and of pap stuffed with raisins, being administered to the unhappy little beings, and wonder how any of them survived. Queen Anne was so habitually over-fed as a child that she had to be sent away at intervals, when she grew too fat, to undergo medical treatment, and this habit of greediness remained with her through life.

The swaddling process inflicted on all high-born children was very rigorously carried out in the case of Royalty. Partly owing to this, and partly owing to the vitiated milk and bad nursing of his foster-mother, James I. was unable to walk when he was five years old, and was always in after life weak on his feet. The amazing rate of infant mortality in former times was largely due to this custom, by which the arms were fastened to the sides, and the legs stretched out and bound together with tight rollers or swaddling bands.

"Baby Charles," afterwards Charles I., suffered cruelly from the same causes as his father. Sir Robert Carey, to whose wife the care of the young Prince was committed when he was between three and four years old, thus describes his state and the drastic methods of cure proposed by the King: "When the little Duke was first delivered to my wife, he was not able to go, nor scarcely to stand alone, he was so weak in his joints, especially in his ankles, insomuch many feared they were out of joint. Many a battle my wife had with the King, but she still prevailed. The King was desirous that the string under his tongue should be cut, for he was so long beginning to speak that he thought he would never have spoken. Then he would have him put in iron boots to strengthen his sinews and joints, but my wife protested so much against them both that she got the victory, and the King was fain to yield." It is satisfactory to know that Lady Carey's treatment was amply justified by the speedy improvement in her little charge.

In the next generation we find the future Charles II. strenuously resisting the efforts of his mother and tutor to physic him. In the first letter (preserved in the British Museum) which the Queen Henrietta Maria writes to her eight year old son, she says: "Charles, I am sorry I must begin my first letter with chiding you because I hear you will not take physicke. I hope it was onlie for this day, and that to-morrow you will do it; for if you will not I must come to you and make you take it, for it is for your health." A letter of the young Prince to his governor, Lord Newcastle, of about the same period, is also in existence, in which he writes in round text hand: "My Lord, I would not have you take too much physicke, for it doth always make me worse, and I think it will doe the like with you. I ride every day, and am ready to follow any other directions from you." It is not very likely that he succeeded in having his own way in the matter of physic or anything else. Those were days of rigorous discipline for children and even kings were not exempt from the dominion of the rod. Going back to the times of the infant Henry VI., we find a quaint order in council, worded as if from the baby king himself, to his governess, Dame Alice, "from time to time reasonably to chastise us, as the case may require, without being held accountable or molested for the same at any future time."

The chastisement was not always reasonable. Childish ailments were very often considered symptoms of obstinacy, and punished as such. There is no instance of this more pitiable than the case of the young Duke of Gloucester, eldest son of Queen Anne. Baby after baby had died of water on the brain, and this last survivor suffered terribly from the same malady. "His hat, poor infant," writes his biographer, Lewis Jenkins, "at five years old was large enough for most men. His complaint seems to have been little understood, because when ever and anon the suffering child craved the assistance of two persons to lead him on each side, especially when he went up and down stairs, his demand of support was treated as idle whim. The Princess shut herself up with her little son for more than an hour, trying to reason with him that it was improper to be led up and down stairs at the age of more than five years; she led him into the middle of the room and told him to walk, as she was sure he could do so. He obstinately refused to stir without being led by at least one person. The Princess then took a birch rod, and gave it to Prince George, who repeatedly slashed his son with it in vain; at last by dint of severe strokes the torture made him run alone." The constant effort on the part of the sickly child to appear robust and manly enough to satisfy his father brought on repeated attacks of illness. Still he struggled on for some three or four years more, bearing up even under the miserable cramming system, by means of which he was able at the age of ten to astonish his examiners by his answers on "jurisprudence, the Gothic laws, and the feudal system." Then he sickened of a fever, and in the absence of Dr. Radcliffe was bled by the physician in attendance. Dr. Radcliffe, on arriving, declared the malady to be scarlet fever, and learning the treatment which had been adopted, bluntly declared, "Then you have destroyed him, and you may finish him, for I will not prescribe." Thus a final blunder completed the series, which had extended over the short suffering life of the Prince, and carried off the last direct heir of the Stuart family.

